# The Utilisation of Antarctic Microalgae Isolated from Paradise Bay (Antarctic Peninsula) in the Bioremediation of Diesel

**DOI:** 10.3390/plants12132536

**Published:** 2023-07-03

**Authors:** Nur Diyanah Zamree, Nurul Aini Puasa, Zheng Syuen Lim, Chiew-Yen Wong, Noor Azmi Shaharuddin, Nur Nadhirah Zakaria, Faradina Merican, Peter Convey, Syahida Ahmad, Hasrizal Shaari, Alyza Azzura Azmi, Siti Aqlima Ahmad, Azham Zulkharnain

**Affiliations:** 1 Department of Biochemistry, Faculty of Biotechnology and Biomolecular Sciences, Universiti Putra Malaysia, Serdang 43400, Selangor, Malaysia; diyanahzamree@gmail.com (N.D.Z.); nurulainipuasa@gmail.com (N.A.P.); syuenylim@gmail.com (Z.S.L.); nadhirahairakaz@gmail.com (N.N.Z.); syahida@upm.edu.my (S.A.); 2 School of Health Sciences, International Medical University, Bukit Jalil 57000, Kuala Lumpur, Malaysia; wongchiewyen@imu.edu.my; 3 School of Biological Sciences, Universiti Sains Malaysia, Minden 11800, Pulau Pinang, Malaysia; faradina@usm.my; 4 British Antarctic Survey, High Cross, Madingley Road, Cambridge CB3 0ET, UK; pcon@bas.ac.uk; 5 Department of Zoology, University of Johannesburg, P.O. Box 524, Auckland Park 2006, South Africa; 6 Millennium Institute Biodiversity of Antarctic and Subantarctic Ecosystems (BASE), Las Palmeras 3425, Ñuñoa 7750000, Santiago, Chile; 7 School of Marine and Environmental Sciences, Universiti Malaysia Terengganu, Kuala Nerus 21030, Terengganu, Malaysia; riz@umt.edu.my; 8 Faculty of Science and Marine Environment, Universiti Malaysia Terengganu, Kuala Nerus 21030, Terengganu, Malaysia; alyza.azzura@umt.edu.my; 9 Laboratory of Bioresource Management, Institute of Tropical Forestry and Forest Products (INTROP), University Putra Malaysia, Serdang 43400, Selangor, Malaysia; 10 Material Synthesis and Characterization Laboratory, Institute of Advanced Technology, Universiti Putra Malaysia, Serdang 43400, Selangor, Malaysia; 11 Department of Bioscience and Engineering, College of Systems Engineering and Science, Shibaura Institute of Technology, 307 Fukasaku, Minumaku, Saitama 337-8570, Japan

**Keywords:** bioremediation, diesel, microalgae, Antarctic

## Abstract

Research has confirmed that the utilisation of Antarctic microorganisms, such as bacteria, yeasts and fungi, in the bioremediation of diesel may provide practical alternative approaches. However, to date there has been very little attention towards Antarctic microalgae as potential hydrocarbon degraders. Therefore, this study focused on the utilisation of an Antarctic microalga in the bioremediation of diesel. The studied microalgal strain was originally obtained from a freshwater ecosystem in Paradise Bay, western Antarctic Peninsula. When analysed in systems with and without aeration, this microalgal strain achieved a higher growth rate under aeration. To maintain the growth of this microalga optimally, a conventional one-factor-at a-time (OFAT) analysis was also conducted. Based on the optimized parameters, algal growth and diesel degradation performance was highest at pH 7.5 with 0.5 mg/L NaCl concentration and 0.5 g/L of NaNO_3_ as a nitrogen source. This currently unidentified microalga flourished in the presence of diesel, with maximum algal cell numbers on day 7 of incubation in the presence of 1% *v*/*v* diesel. Chlorophyll *a*, *b* and carotenoid contents of the culture were greatest on day 9 of incubation. The diesel degradation achieved was 64.5% of the original concentration after 9 days. Gas chromatography analysis showed the complete mineralisation of C_7_–C_13_ hydrocarbon chains. Fourier transform infrared spectroscopy analysis confirmed that strain WCY_AQ5_3 fully degraded the hydrocarbon with bioabsorption of the products. Morphological and molecular analyses suggested that this spherical, single-celled green microalga was a member of the genus *Micractinium*. The data obtained confirm that this microalga is a suitable candidate for further research into the degradation of diesel in Antarctica.

## 1. Introduction

Antarctica is a unique and pristine environment that is a focus of scientific research, and key in the conservation of global biodiversity. It hosts a diverse array of charismatic species, including penguins, seals and whales [1]. However, this fragile environment is facing a number of threats, including the occurrence of oil spills [2]. One of the largest oil spill incidents to date involved the sinking of the Argentinian supply vessel Bahía Paraíso in 1989. This resulted in 600,000 L of diesel being released into the sea and coastline in Arthur Harbour, close to Palmer Station, Anvers Island, western Antarctic Peninsula [3].

Diesel is currently the primary source of energy supporting human activities in Antarctica, such as automotive fuel supply and electricity generation for research stations [4]. However, if accidentally released into the environment, diesel can be damaging and detrimental, due to the release of harmful pollutants such as polycyclic aromatic hydrocarbons (PAHs) and sulphur compounds [5]. Antarctica’s high latitude location in the Southern Hemisphere results in it having an extreme climate [6]. Its chronically low temperatures limit the degradation of diesel contaminants, which persist in the environment over longer periods, in part due to increased viscosity [7], leading to further negative impacts on the local ecosystem.

Given the potential consequences of diesel pollution, there have been increasing research efforts towards the clean-up of hydrocarbon pollutants [8]. Bioremediation has been shown to be a successful strategy. This method of clean-up has been efficient in removing 60% of hydrocarbon contaminants from polluted soils near Carlini Station, King George Island, using bacteria such as *Pseudomonas* sp. [9]. To date, bioremediation approaches examined in Antarctica have focused on the use of bacteria, fungi and yeasts [10,11]. However, it has been suggested that a variation in bioremediation or phytoremediation has significant potential in the remediation of petrogenic hydrocarbon-contaminated soil [12]. Yet, the application of phytoremediation by photosynthetic microorganisms in Antarctica remains to be explored.

Chlorophyta are green microalgae found in terrestrial, marine and freshwater environments [13]. In comparison with higher plants, algae posses superior photosynthetic efficiency [14], associated with an increased ability for aerobic breakdown of organic compounds. Based on research by García de Llasera et al. [15], the potential of microalgae for use as phytoremediators was confirmed by *Selenastrum capricornutum* and *Scenedesmus acutus*. These species of freshwater microalgae from Austin, Texas, showed great capability to degrade PAHs.

The implementation of microalgae in the phytoremediation of pollutants present in Antarctica has received little attention to date. Antarctic microalgae have also demonstrated a high tolerance of exposure to hydrocarbons such as Special Antarctic Blend (SAB) diesel, in apparent contrast with the presumption that the native microbial communities of Antarctica are highly sensitive to contaminants [16]. Studies have yet to attempt to utilise Antarctic microalgae as hydrocarbon degraders [4]. Therefore, this study set out to test whether a newly isolated microalga obtained from Paradise Bay, western Antarctic Peninsula, could successfully degrade diesel at low temperatures mimicking those of the Antarctic environment.

## 2. Results and Discussion

### 2.1. Algal Growth Analysis of Isolate WCY_AQ5_3

**Figure 1:**
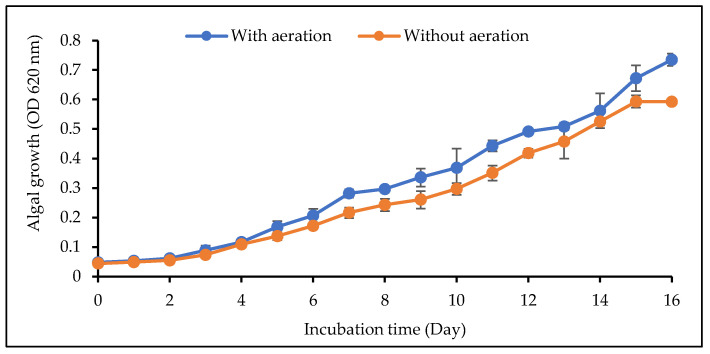
Semi-logarithmic growth curve based on OD 620 nm of the studied algal isolate in different systems. The culture was grown in BBM for 16 days. Error bars represent the mean ± SEM (*n* = 3).

The WCY_AQ5_3 isolate was subjected to two different cultivation systems, aerated and non-aerated. As reported by Barsanti and Gualtieri [17], microalgae typically undergo several growth phases—lag, exponential, deceleration, stationary and death. During the initial incubation period up to the second incubation day, the microalgae were in the lag phase (Figure 1). From day 4, exponential growth was observed in both systems. On day 15, the contrasting growth patterns of the microalgae in the two systems became apparent. Without aeration, isolate WCY_AQ5_3 entered the stationary phase, whilst the aerated microalgae continued to grow steadily, with no signs of deceleration until the end of the incubation period. Similarly, Ugwu et al. [18] established that the slow growth rate of microalgae in a non-aerated system was due to the algal cells settling in the bottom of the flask where, over time, some cells are not able to obtain sufficient light and nutrients. The statistical analysis demonstrated that the algal growths of aerated and non-aerated samples were suggested as significant, at *p* < 0.0001, F (16, 34) = 63.07 and *p* < 0.0001, F (16, 34) = 85.36, respectively.

### 2.2. Optimisation of Algal Growth and Diesel Degradation Using One-Factor-at-a-Time (OFAT)

**Figure 2:**
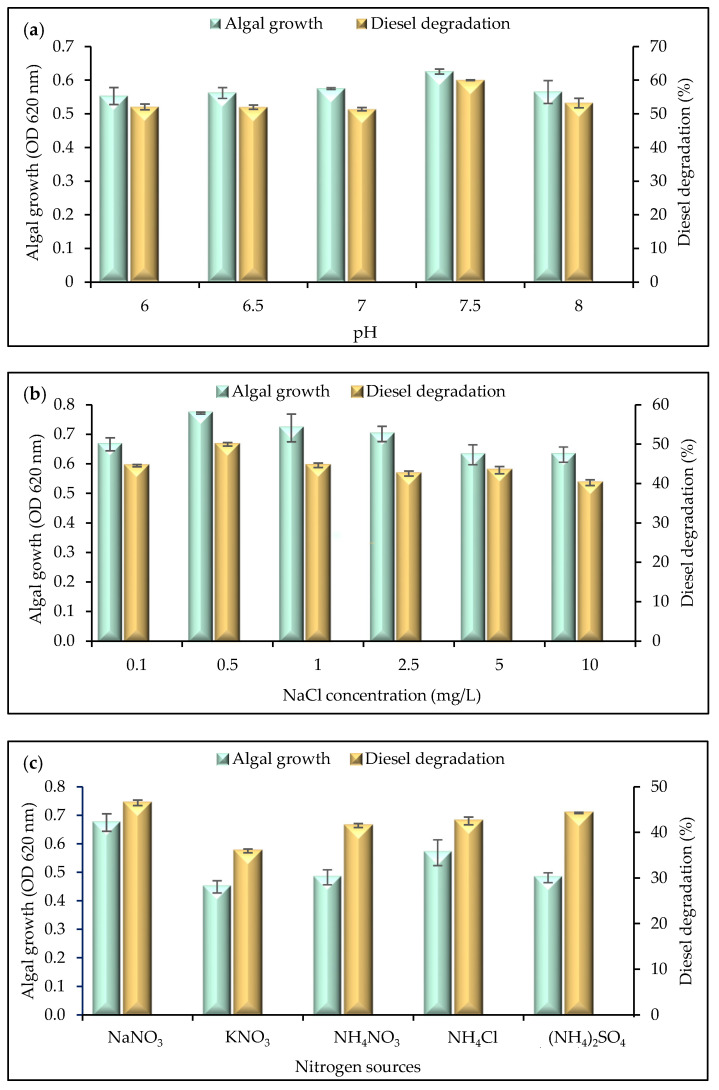

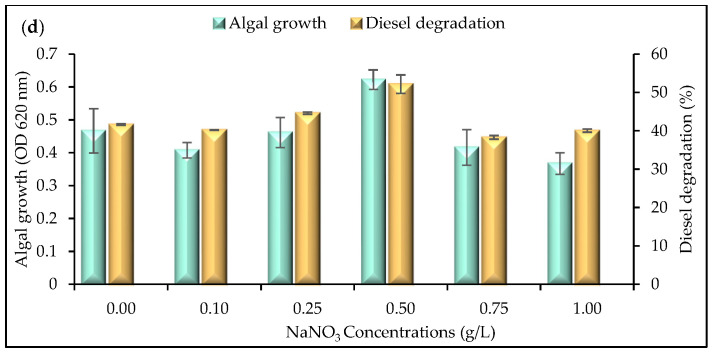
Effects of (**a**) pH, (**b**) salinity, (**c**) nitrogen sources and (**d**) selected nitrogen source (NaNO_3_) concentration on the growth and diesel degradation performance of isolate WCY_AQ5_3. Error bars represent the mean ± SEM (*n* = 3).

The growth of microalgae is greatly influenced by the pH of their environment. The pH of cultivation media can affect processes such as enzymatic and metabolic activities. Alkaline conditions were preferred by isolate WCY_AQ5_3, under which it showed the highest growth and diesel degradation performance (Figure 2a). In a study investigating green Antarctic snow algae, the optimum pH for the *Chloromonas chenangoensis* strain CU 722B was 7.0–8.0. Similarly, another snow microalga, *Chlamydomonas applanate* demonstrated an optimal pH of 7.4 [19]. However, a number of non-Antarctic strains of *Chlorella vuglaris* species have a neutral optimal pH of 7.0 [20,21]. The statistical analysis confirmed the significant effect of pH on microalga diesel degradation (*p* = 0.0151, F (5, 12) = 4.517); however, the growth was found to be not significant (*p* = 0.3901, F (5, 12) = 1.143).

To achieve optimal microalgae growth, it is important to consider the limited tolerance of freshwater microalgae towards extreme salinity conditions [22]. Isolate WCY_AQ5_3 showed highest growth and diesel degradation performance when 0.5 mg/L of NaCl was present (Figure 2b). It is also evident that, as the NaCl concentration was increased further, the algal growth and diesel degradation performance reduced, consistent with this strain being a freshwater isolate. High salinity affects the cell division and motility of green microalgae [23,24,25,26], as specifically demonstrated in *Chlorella vulgaris*, *C. salina*, *C. emersonii* [27] and *Scenedesmus opoliensis* [28]. The statistical analysis found a significant effect of salinity on microalga diesel degradation (*p* = 0.0124, F (5, 12) = 4.779); however, the growth was found to be not significant (*p* = 0.2221, F (5, 12) = 1.645).

In the cultivation of microalgae, a source of nitrogen is required as a macronutrient. Nitrogen accelerates growth and contributes to the regulation of metabolic activities, such as lipid, fatty acid and carbohydrate synthesis [29,30,31,32]. Nitrogen can be assimilated by microalgae as nitrate, nitrite, urea and ammonium. The nitrogen source that led to the highest algal growth and diesel degradation performance in this study was sodium nitrate, NaNO_3_. Although ammonium can be used as an important nitrogen source in some algal culture studies, it was likely not a favoured source of nitrogen here due to causing a pH shift, as suggested by Procházková et al. [33]. According to statistical analysis, the nitrogen source significantly affected the degradation and growth of microalga (*p* = 0.0019, F (4, 10) = 9.524 and *p* = 0.0003, F (4, 10) = 15.11), respectively.

The concentration of nitrogen is also important to consider when investigating algal growth and diesel degradation performance. Nitrogen source concentrations can influence the biochemical composition and growth of microalgae [34]. In the current study, a nitrogen concentration of 0.5 g/L yielded the highest algal growth, and up to 52.16% diesel degradation. This is consistent with the study of Daliry et al. [35], who reported that a similar species of microalga grew optimally with 0.5 g/L of potassium nitrate (KNO_3_). Based on statistical analysis, nitrogen concentration significantly affected the degradation and growth of microalga (*p* = 0.0180, F (5, 12) = 4.294 and *p* = 0.0193, F (5, 12) = 4.206), respectively.

### 2.3. Comparing the Growth of Microalgae with and without the Presence of Diesel

**Figure 3:**
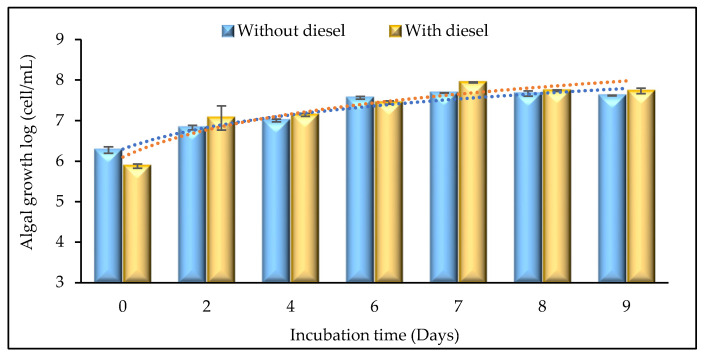
Algal growth in culture in the presence and absence of diesel. Microalgal cultures were incubated for 9 days at 10 °C under the illumination of a cool white fluorescent light (42 µmol/m^2^/s) and a 12:12 h light dark cycle. Error bars represent mean ± SEM (*n* = 3).

Isolate WCY_AQ5_3 was cultured to determine experimentally its ability to grow under exposure to diesel. Figure 3 illustrates algal growth in the absence or presence of diesel over a 9-day incubation. Algal cell counts were logarithmically transformed to assist in identifying the phases of the growth curve. In the absence of diesel, algal growth increased until day 6. On days 7 to 9 growth entered the lag phase. In the presence of diesel, algal growth peaked on day 7, after which it stabilised. Comparing the two conditions, the microalgae appeared to perform slightly, though not significantly, better in the presence of diesel. Maximum cell density was seen on day 7, with over 87 million cells/mL. Yap et al. [36] noted that the hydrocarbon constituents in diesel can act as a carbon source for microalgal respiration and growth.

**Figure 4:**
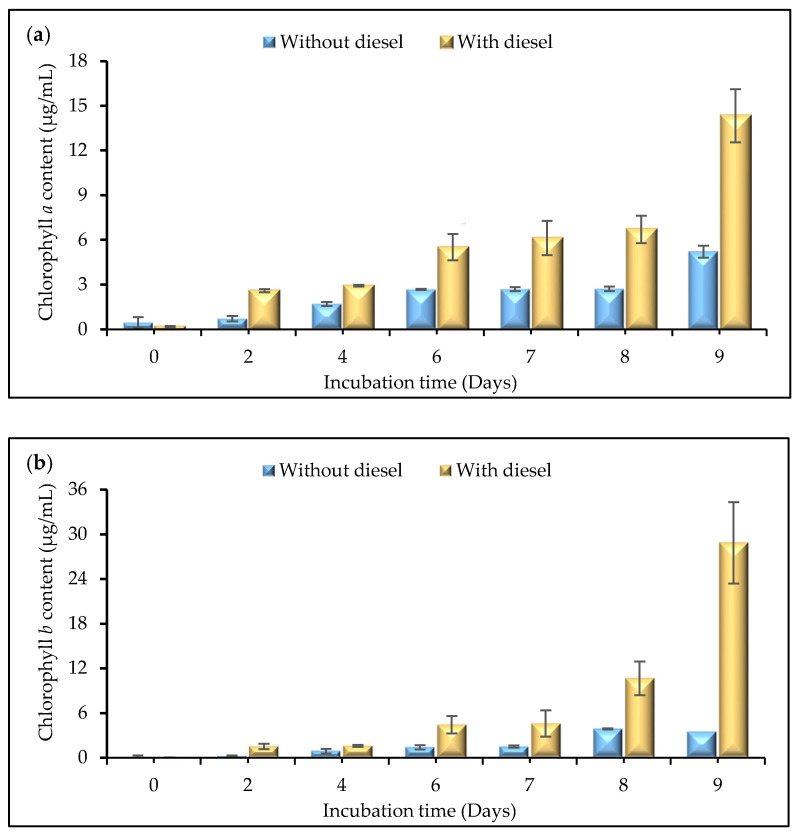

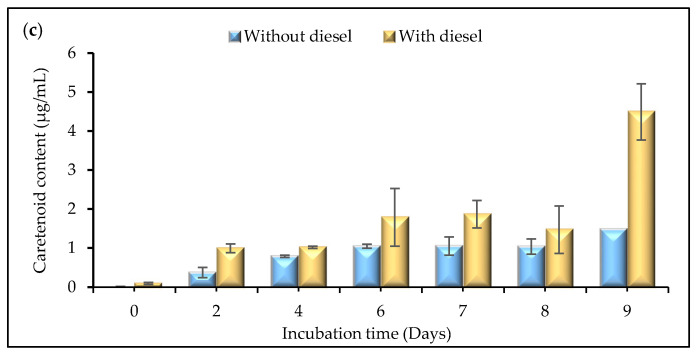
Photosynthetic pigment contents of hydrocarbon-degrading microalgae over a 9-day incubation period in either the absence or presence of 1% *v*/*v* diesel; (**a**) chlorophyll *a* content, (**b**) chlorophyll *b* content, (**c**) carotenoid content. Error bars represent mean ± SEM (*n* = 3).

Photopigment analysis was carried out to determine the concentrations of chlorophyll *a*, *b* and carotenoids in the microalgal culture. Chlorophyll *a* and chlorophyll *b* are primary pigments responsible for photosynthesis in microalgae, and give chlorophyte cultures their green colour [37]. The amount of these pigments present indicates the growth and biomass production of the culture. Carotenoids, on the other hand, are accessory pigments that play a role in mitigating reactive oxygen species (ROS), and serve as antioxidants, protecting the cells against oxidative damage. As shown in Figure 4a, the chlorophyll *a* content gradually increased in both the presence and absence of diesel throughout the 9 days of incubation. However, chlorophyll *a* content was significantly higher in the presence of 1% *v*/*v* diesel. Again, this is consistent with the diesel acting as a carbon source for the microalgae. Similarly, as shown in Figure 4b,c, chlorophyll *b* and carotenoid contents were also higher in the presence of diesel. Wood et al. [37] suggested that increased carotenoid content could be the result of a negative feedback to protect cells from damage caused by the diesel.

**Figure 5:**
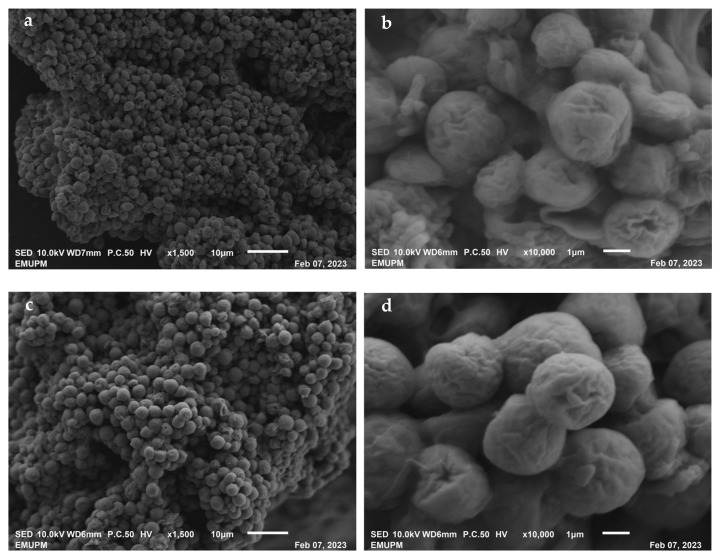
SEM images of microalgal samples cultured in the absence (**a**,**b**) and presence (**c**,**d**) of diesel.

SEM images of microalgal samples cultured in the absence or presence of diesel are shown in Figure 5. These demonstrate that the surface texture of the microalgae remained unaffected after 7 days of incubation without diesel (Figure 5a,b) or with diesel (Figure 5c,d), further supporting that the presence of diesel did not have direct negative impacts on algal growth. According to Markao et al. [38], microalgae need high concentrations of vital nutrients, such as carbon, to achieve optimal growth. This outcome is consistent with previous studies reporting enhanced capacity of microalgae to grow when provided with exogenous carbon sources, such as sucrose or glucose [39,40,41,42].

### 2.4. Diesel Degradation Ability of Microalgae

**Figure 6:**
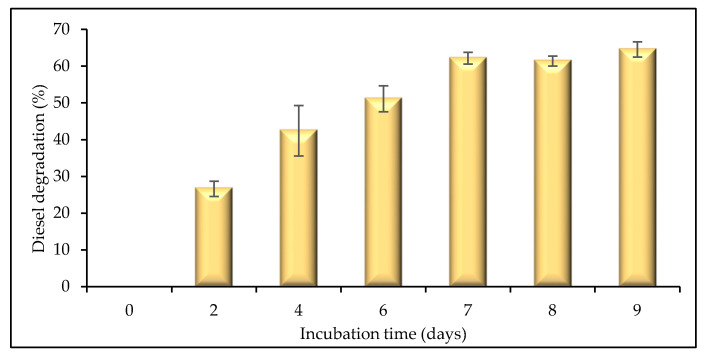
Biodegradation of an initial 1% *v*/*v* diesel concentration achieved by Antarctic microalgal strain WCY_AQ5_3 assessed by gravimetric analysis over a 9 day incubation period.

In order to determine the ability of microalgae to degrade 1% *v*/*v* diesel, gravimetric analysis was carried out (Figure 6). Over the 9 days of incubation, biodegradation of diesel started on day 2, with 26.6% degradation achieved. Total biodegradation increased consistently over the following days until day 7, when degradation started to plateau. The greatest total degradation was recorded on day 9 (64.5%). Das and Deka [43], in a study of the tropical green microalgal strain *Chlorella vulgaris* BS1, reported petroleum degradation of 98.63% over 14 days of cultivation. Similarly, Kalhor et al. [44] reported that *C. vulgaris* showed high potential in degrading as much as 94% of an initial 1% *v*/*v* concentration of crude oil within 7 days, illustrating that microbial degradation accelerates at a higher temperature. Under Antarctic conditions, the low temperatures may result in the inactivation of transport channels in cells and reduce the rates of metabolic processes, hence reducing biodegradation potential [45].

#### 2.4.1. Gas Chromatography Analysis

**Figure 7:**
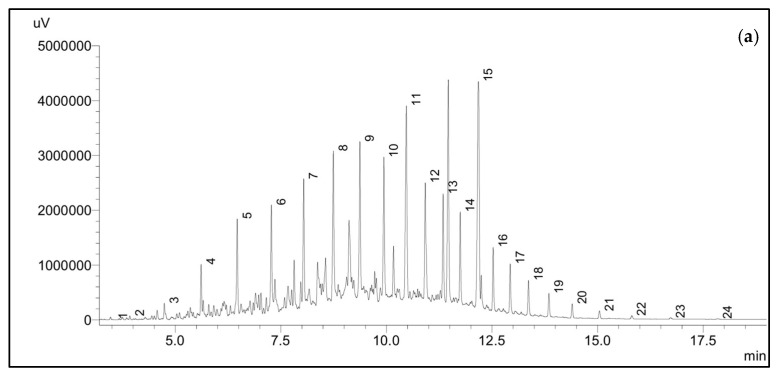

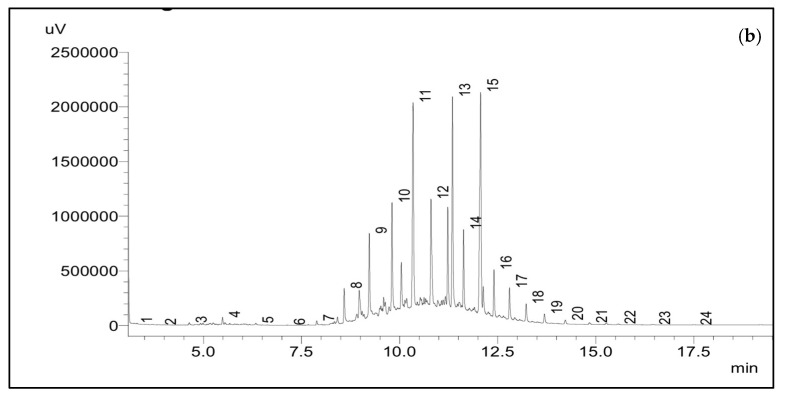
GC-FID profiles of diesel degradation by Antarctic microalgal strain WCY_AQ5_3. (**a**) Profile at initiation (day 0) of incubation period, (**b**) profile at day 9 of incubation period. The internal standards were alkanes of C_7_-C_30_. Alkane compounds are denoted by 1: C_7_; 2: C_8_; 3: C_9_; 4: C_10_; 5: C_11_; 6: C_12_; 7: C_13_; 8: C_14_; 9: C_15_; 10: C_16_; 11: C _17_; 12: C_18_; 13: C_19_; 14: C_20_; 15: C_21_; 16: C_22_; 17: C_23_; 18: C_24_; 19: C_25_; 21: C_26_; 21: C_27_; 22: C_28_; 23: C_29_; and 24: C_30_.

The GC-FID profiles of the analysis of the microalgal degradation of 1% *v*/*v* diesel are shown in Figure 7. Clear reductions in certain peaks are apparent in the chromatogram, supporting the degradation of linear alkanes by strain WCY_AQ5_3, and consistent with the gravimetric analysis results. Figure 7a illustrates the contents of n-alkanes on the initial day of cultivation (day 0). On the final day of incubation (day 9) (Figure 7b) the C_7_–C_13_ alkanes had been completely mineralised. However, longer alkanes (e.g., C_19_) showed much lower proportions of degradation.

Based on the study of Zhang et al. [46], pristane (C_19_) is a hydrocarbon that is composed of a complex branched structure. Due to this, the degradation of pristane is difficult. Some other microorganisms are capable of degrading pristane, such as *Enterobacter* sp. [47], but strain WCY_AQ5_3 does not have this ability. However, it is important to note that the diesel most commonly utilised in Antarctica, Special Antarctic Blend (SAB), predominantly consists of C_9_-C_14_ alkanes [48]. Therefore, strain WCY_AQ5_3 could have good potential in mitigating SAB-sourced hydrocarbon pollution in Antarctica.

#### 2.4.2. FTIR Analysis

**Figure 8:**
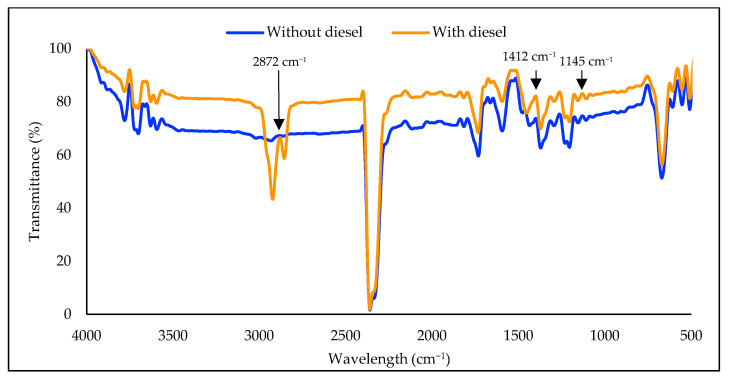
FTIR spectrum of microalgal strain WCY_AQ5_3 in the presence or absence of diesel. The appearance of peaks in the microalgal sample containing diesel supports the absorption of diesel alkanes on to the cell surface (arrows).

The molecular composition and functional groups can be analysed by evaluating the position, width, and strength of infrared light absorption. Figure 8 shows the changes in cell surface functional groups of strain WCY_AQ5_3 when exposed to diesel, and in its absence. The various peaks observed in the FTIR spectra reflect the presence of carbonyl, hydroxyl, carboxylic and amino groups. When diesel is present, peaks were observed at 2983, 2872, 1412 and 1145 cm^−1^, which indicate the stimulation of lipid groups and proteins present on the surface of the cell. Along with band assignments to biomolecules, new peaks at 2872 and 1412 cm^−1^ (indicated by arrows in Figure 8) demonstrate diesel adsorption onto the surface of the algal cells. Qasim et al. [49] reported absorption peaks of diesel alkanes at 2922, 2852 and 1459 cm^−1^. These data indicate that diesel adsorption onto the surface of algal cells has taken place.

### 2.5. Identification of Diesel-Degrading Microalgae

**Figure 9:**
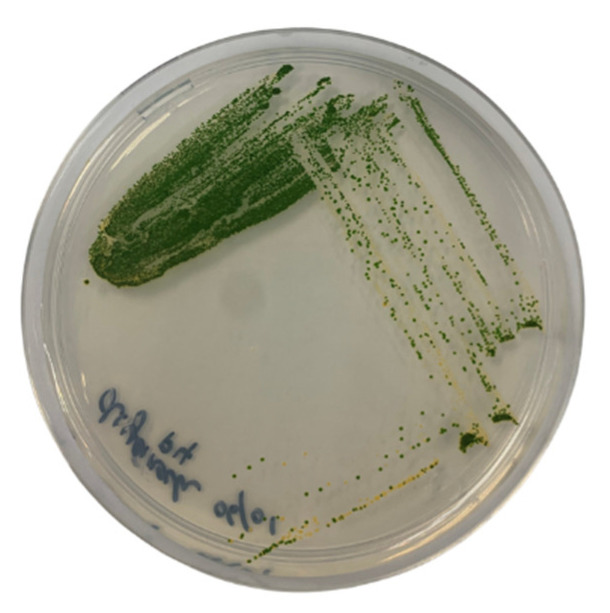
Growth of strain WCY_AQ5_3 on nutrient agar after 12 days of incubation.

**Figure 10:**
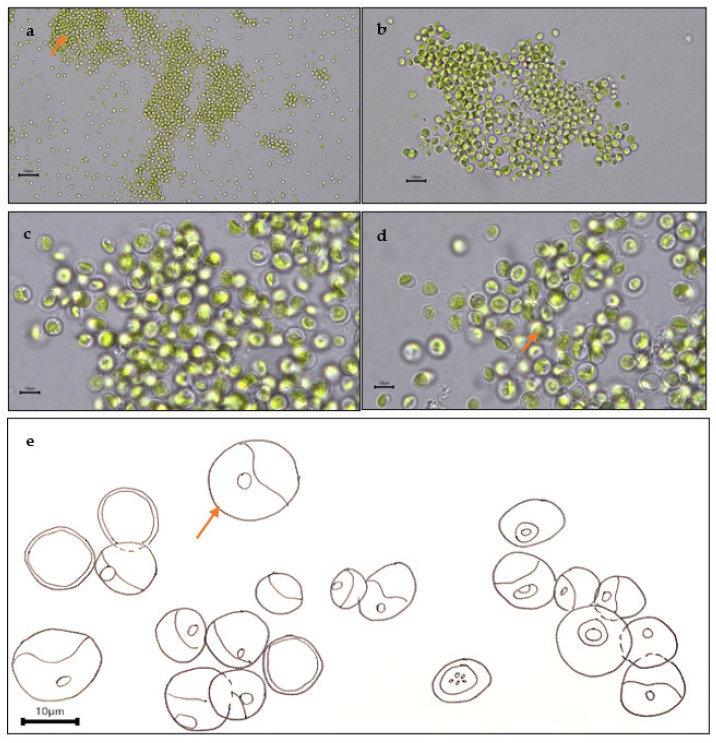
Micrograph images of strain WCY_AQ5_3 grown under typical cultivation conditions at the following magnifications: (**a**) 200×, (**b**) 400×, (**c**,**d**) 2000× and (**e**) 2000×. (**a**) Dense green colonies of spherical cells, (**b**) cells overlapping in a tight bundle (arrow), (**c**) cells containing cup-shaped chloroplasts with one distinct pyrenoid, (**d**) cells possessing a single nucleus (arrow), (**e**) taxonomic illustration depicting the actual size of the microalgae.

Bold’s basal agar (BBA) is a freshwater medium commonly used to cultivate an array of green microalgae. As shown in Figure 9, the culture on the BBA spread plate formed green-coloured colonies. The colonies had a circular form and were opaque. The colonies were flat and not raised [50]. To describe the morphological characteristics of strain WCY_AQ5_3, preliminary observation was carried out using light microscopy. Figure 10 shows four images of strain WCY_AQ5_3. Cells were spherical with diameter of 2–4 µm.

The bright grass-green cells were arranged densely with cells overlapping, occasionally occurring as solitary cells (Figure 10a). Individual cells contained a distinct cup-to-girdle shaped chloroplast with a spherical to ellipsoidal pyrenoid (Figure 10e). The hyaline sheath was thin, surrounding the cells, and the nucleus was present at the centre of the cell (Figure 10c,d). These characteristics are consistent with previous studies of the genus *Chlorella*, which has spherical-ovoid cells that also have the presence of a pyrenoid with parietal cup-shaped chloroplast [51].

**Figure 11:**
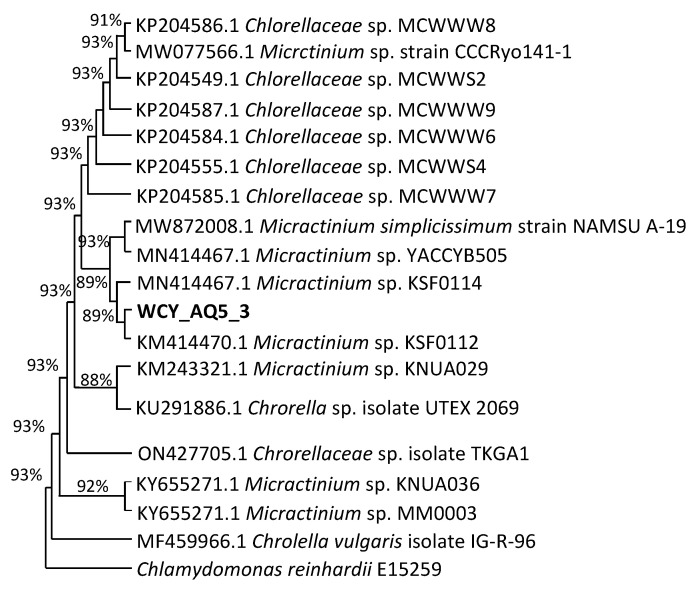
Maximum likelihood phylogenetic tree of the consensus ITS sequence of microalgal strain WCY_AQ5_3 using MegaX v.11 with bootstrap values indicated.

Coccoid green algae such as *Chlorella* have very simple morphologies. Therefore, one highly variable genetic locus, such as ITS, can be sufficient for their identification [52,53]. With the application of Mega X, a phylogenetic tree was constructed to illustrate the relationships between closely related taxa using the maximum likelihood approach. As noted above, microalgal isolate WCY_AQ5_3 superficially resembles the genus *Chlorella* morphologically, due to its single cell features and the presence of a pyrenoid. However, molecular evaluation placed the strain in the clade containing *Micractinium*, suggesting a close relationship of the strain to this genus. Figure 11 displays the affiliation of strain WCY_AQ5_3 with 18 closely related members of the family Chlorellaceae, with *Chalamydomonas reinhardii* E15259 as the outgroup. Analysis of the strain’s ITS sequence revealed that it shared the highest similarity with *Micractinium* sp. KNUA036 (92%), *Micractinium* sp. KSF0114 (89%), *Micractinium* sp. KSF0112 (89%) and *Micractinium* sp. KNUA029 (88%). This result does not support strain WCY_AQ5_3 being placed in *Chlorella*, rather being a representative of *Micractinium*. Microalgae representing *Chlorella* and *Micractinium* are closely similar morphologically [54]. The distinctive features separating these genera are the presence of spines or bristles in most *Micractinium* sp. However, strain WCY_AQ5_3 had neither spines not bristles. Hong et al. [55] confirmed that not all *Micractinium* sp. have bristles and spines. Other *Micractinium* sp. with close relationship to strain WCY_AQ5_3 included cold-tolerant microalgae isolated from Antarctic freshwater obtained from Deception Island and King George Island [56], which are both in the South Shetland Islands north-west of the Antarctic Peninsula, and relatively close to the current study location. The outcome of this molecular analysis confirms the importance of molecular evaluation. Due to the oversimplified and minute characteristics of microalgae, distinctive features may not be visible under a microscope. Misidentification in morphological analysis can also be influenced by the stage of the cells examined. Hence, it is important to observe mature cells in a proper cultivation setting in terms of temperature, light and nutrient media [17].

## 3. Materials and Methods

### 3.1. Source of Microalgae and Diesel

The unidentified freshwater microalgal isolate P4 was originally isolated from Paradise Bay, western Antarctic Peninsula (S 64°51′, W 62°55′). It was provided by the School of Health Sciences, International Medical University (IMU), Kuala Lumpur, Malaysia. Diesel was sourced from PETRONAS Dynamic Diesel Euro 5 from Petronas Sri Serdang (NGV Petronas UPM Serdang), Seri Kembangan, Selangor.

### 3.2. Screening Growth of Microalgae

Fifty microlitres of microalgal source culture was cultivated in 250 mL sterile conical flasks containing 100 mL BBM with or without aeration. BBM contained, per litre, 0.25 g NaNO_3_, 0.075 g MgSO_4_.7H_2_O, 0.12 g K_2_HPO_4_, 0.15 g KH_2_PO_4_, 0.025 g CaCl_2_, 0.025 g NaCl, 0.0114 g H_3_BO_4_, 0.05 g EDTA.Na_2_, 0.031 g KOH, 0.00498 g FeSO_4_, 0.001 mL concentrated HCl, 0.00882 g ZnSO_4_, 0.00071 g MoO_3_, 0.00049 g Co(NO_3_)_2_.6H_2_O, 0.00144 g MnCl_2_, and 0.00157 g CuSO_4_.5H_2_O. The flasks were incubated at 10 °C under the illumination of a cool white fluorescent lamp of photon flux 42 µmol m^−2^s^−1^, with a 12:12 h light-dark cycle. Subsequently, the cell density of algae was standardised at OD_620nm_ (0.1 ± 0.1) by adjusting the amount of BBM and microalgae using a UV-Vis spectrophotometer [57].

### 3.3. Optimisation of Algal Growth and Diesel Degradation Using One-Factor-at-a-Time (OFAT)

Algal growth and diesel degradation were optimised using the OFAT methodology applied with the following parameters: pH (6, 6.5, 7, 7.5 and 8), salinity (0.1, 0.5, 1.0, 2.5, 5 and 10 mg/L of NaCl), nitrogen source (NaNO_3_, KNO_3_, NH_4_NO_3_, NH_4_Cl (NH_4_)_2_SO_4_ and nitrogen concentration (0, 0.1, 0.25, 0.50, 0.75 and 1.00 g/L of NaNO_3_). The statistical analysis was examined using a one-way variance analysis (ANOVA) in the GraphPad Prism 8.0.2 programme (GraphPad Inc., San Diego, CA, USA) [58].

### 3.4. Comparing Microalgal Growth

Microalgal growth was assessed in the presence or absence of diesel. Assessment took place on days 0, 2, 4, 6, 7, 8 and 9 of the study. Two millilitres of microalgal culture were added into each of six microcentrifuge tubes. The microalgal samples were obtained from three different flasks containing the sample and diesel. Three of the microcentrifuge tubes were used for cell counts using a hemacytometer. Counting of cells was carried out with the aid of a light microscope [59], while the other three tubes were used for photosynthetic pigment analyses. Chlorophyll *a*, chlorophyll *b* and carotenoid readings at 470, 652 and 665 nm were recorded for each sample using a UV-Vis spectrophotometer (Jen Way, Stone, UK) [60].

### 3.5. Screening the Diesel Degrading Abilities of Microalgae (1% v/v)

Diesel degradation efficiency of the microalgal strain was assessed by performing gravimetric analysis from days 0–9 using the formula below [61].

(1) $$\mathrm{BE}\left(\%\right)=100\times \frac{\left(\mathrm{Original}\;\mathrm{mass}\;\mathrm{of}\;\mathrm{oil}-\mathrm{mass}\;\mathrm{of}\;\mathrm{final}\;\mathrm{residual}\;\mathrm{oil}\right)}{\mathrm{Original}\;\mathrm{mass}\;\mathrm{of}\;\mathrm{oil}\;\mathrm{introduced}}$$

To provide a preliminary analysis of the diesel degradation and functional groups present on the cell surface, gas chromatography and Fourier-transform infrared spectroscopy (FTIR) analyses were conducted on algal samples before and after exposure to 1% *v*/*v* diesel from the day 7 gravimetric analysis. Analysis of residual hydrocarbons present in the extracted diesel was carried out using gas chromatography (GC-2010 Plus Shimadzu). The gas chromatograph equipped with an HP-5ms capillary column (30 m × 0.25 mm × 0.25 µm) supplied with a flame ioniser detector (FID) and auto-sampler (AOC-20i) was used, while, for the FTIR analysis, the algal cell pellets were collected and freeze-dried for 48 h before the analysis. Through the attenuated total reflection (ATR) mechanism, the infrared absorption spectra were scanned between 4000 and 400 cm^−1^, with the aid of the FTIR (ALPHA, Bruker Optik GmbH, Ettlingen, Germany).

### 3.6. Identification

The microalgal strain was identified using morphological and molecular analyses. To study the diacritical features of the green microalgae, morphological analyses were carried out by plate streaking and light microscopy. For molecular analysis, genomic DNA extraction of the microalgal sample was carried using the Machery Nagel NucleoSpin Plant II kit before subjecting the products it to polymerase chain reaction (PCR) and analysis of the internal transcribed spacer (ITS) region. The polymerase chain reaction (PCR; Thermal Cycler, Bio-Rad Laboratories, Hercules, CA, USA) was carried out to amplify the extracted gDNA. This was accomplished by the utilisation of the ITS primers ITS 1 (5′-TCC GTA GGT GAA CCT GCG G-3′) and ITS 4 (5′-TCC TCC GCT TAT TGA TAT GC-3′). Thermal cycling conditions were set at 94 °C for 2 min for pre-denaturation before 35 cycles of denaturation at 94 °C for 30 s, annealing at 54 °C for 30 s and 72 °C for 30 s, with a final extension at 72 °C for 7 min. The quality of the PCR product was validated using 1% agarose gel in 1× Trisacetate-EDTA (TAE) buffer before it was submitted for Sanger sequencing by NextGene Scientific Sdn. Bhd. MEGA X software version 11 was used to construct a phylogenetic tree, using 1000 bootstrap replications, with *Chlamydomonas reinhadii* E15259 used as the outgroup [62,63].

## 4. Conclusions

Microalgal strain WCY_AQ5_3, originally obtained from Paradise Bay, north-west Antarctic Peninsula, showed high potential in the degradation of 1% *v*/*v* Malaysian-sourced diesel. Its considerable tolerance towards hydrocarbon exposure suggests it can be considered as a suitable instrument in the development of new phytoremediation approaches suitable for the Antarctic environment. In comparison of microalgal growth in the presence and absence of diesel, strain WCY_AQ5_3 grew well and without apparent negative effects over 9 days of incubation at 10 °C in diesel-containing medium. Contents of chlorophyll *a*, *b* and carotenoids contents were increased in algae exposed to diesel, further illustrating their tolerance of this challenge. Strain WCY_AQ5_3 was able to degrade up to 64.5% of the initial 1% *v*/*v* diesel within 9 days in culture, with complete removal of C_9_–C_13_ alkanes as confirmed by gas chromatography analysis. Using morphological and molecular biological analyses, strain WCY_AQ5_3 was confirmed to be a member of the genus *Micractinium*. The outcomes of this study supported the hypothesis that strain WCY_AQ5_3 has the ability to successfully degrade diesel at cold temperatures via phytoremediation. Upon the findings of this investigation, we recommend further exploration of the metabolic processes exhibited by the WCY_AQ5_3 strain in future research. In addition, conducting studies on the degradation abilities of this microalgae strain across various diesel compositions holds significant potential for valuable insights.

## Data Availability

Not applicable.
